# Molecular mechanisms and therapeutic strategies of immunotherapy resistance in esophageal cancer

**DOI:** 10.3389/fimmu.2026.1973515

**Published:** 2026-10-06

**Authors:** Yiqian Pang, Panpan Jiang, Ke Liu, Xingyao Suo, Dianbao Zhang, Yishi Xu, Yi Ge, Qizhi Fu, Shegan Gao

**Affiliations:** 1 Key Laboratory of Microbiome and Esophageal Cancer Prevention and Treatment, The First Affiliated Hospital, and College of Clinical Medicine of Henan University of Science and Technology, Luoyang, China; 2 Institute of Organoid on Chip and Drug Translation Research, Henan Academy of Sciences, Zhengzhou, China

**Keywords:** combination therapy, esophageal cancer, immunotherapy resistance, metabolic reprogramming, tumor microenvironment

## Abstract

Immunotherapy, particularly immune checkpoint inhibitors (ICIs), has transformed the treatment landscape for advanced esophageal cancer (EC). Nevertheless, clinical benefits are constrained by primary and acquired resistance driven by a dynamic immunosuppressive tumor microenvironment (TME), extensive metabolic reprogramming, and epigenetic dysregulation. This review provides a comprehensive analysis of the molecular mechanisms underlying immunotherapy resistance in EC, including TME remodeling by cancer-associated fibroblasts, tumor-associated macrophages, and immunosuppressive cell populations; metabolic reprogramming encompassing glucose, amino acid, and lipid metabolism; epigenetic regulation involving DNA methylation, histone modifications, and non-coding RNAs; and compensatory immune checkpoint upregulation. We further highlight the emerging role of the microbiome and critically evaluate combination strategies designed to overcome resistance, including anti-angiogenic agents, ADCs, and personalized approaches guided by multi-omics and organoid models.

## 1 Introduction

Esophageal cancer ranks as one of the most prevalent and lethal malignant tumors of the digestive tract globally, occupying the eighth position in cancer-related mortality rates (1–3). The two predominant histological subtypes are squamous cell carcinoma (ESCC) and adenocarcinoma (EAC). In China, the predominant histological subtype is ESCC (4, 5). Despite considerable advancements in multidisciplinary treatment approaches, encompassing surgery, chemotherapy, and radiotherapy, the prognosis for patients with EC, particularly in advanced stages, remains poor, characterized by a low 5-year survival rate. The high invasiveness of EC, coupled with its propensity for rapid progression and metastasis, highlights the urgent need for more effective and durable therapies (6, 7).

In recent years, the advent of immunotherapy, particularly the utilization of ICIs, has significantly transformed the landscape of cancer treatment (8). These groundbreaking agents, including anti-programmed cell death protein 1 (PD-1) and anti-programmed cell death ligand 1 (PD-L1) monoclonal antibodies, have shown considerable clinical benefits across various malignant tumors, notably in advanced EC, thereby offering renewed hope to patients. Currently, several immunotherapeutic agents, such as pembrolizumab and nivolumab, have received approval for the treatment of advanced EC (9–13). Nevertheless, despite these advancements, the overall response rate (ORR) among ESCC patients undergoing immunotherapy remains relatively low, with a significant proportion of patients either displaying primary resistance or subsequently developing acquired resistance over time (14–16). This issue of drug resistance poses a substantial challenge to the long-term efficacy of ICIs, underscoring the necessity for a comprehensive understanding of the molecular mechanisms that underpin immune escape and treatment failure in esophageal cancer.

Immune escape in ESCC arises from the dynamic interplay between tumor cells, their microenvironment, and intrinsic cellular regulatory networks (17–19). A deeper understanding of these mechanisms is crucial for developing effective combination therapies and predictive biomarkers to improve patient outcomes. This review aims to systematically analyze the fundamental mechanisms of ICI resistance in EC, focusing on TME remodeling, metabolic reprogramming, and the roles of non-coding RNAs and epigenetic regulation. By synthesizing recent insights from single-cell sequencing, multi-omics analyses, and clinical studies, we endeavor to establish a coherent framework for understanding immune resistance. Given the global epidemiological differences, this review primarily focuses on ESCC, which is predominant in East Asia, while also incorporating key findings from EAC where relevant to highlight both shared and distinct resistance mechanisms. We aim to propose innovative combinatorial strategies to overcome this major clinical challenge. Whereas prior reviews have largely addressed individual axes of immunotherapy resistance in esophageal cancer in isolation—such as immune checkpoint signaling or TME composition—the present review is distinguished by an integrative, mechanism-centered framework. Specifically, we position the cross-regulation between metabolic reprogramming and epigenetic modifications as a central organizing principle, incorporate the emerging contribution of the tumor microbiome, and synthesize recent single-cell and multi-omics evidence, thereby connecting each resistance mechanism to mechanism-guided combination strategies. Throughout the review, we further distinguish the mechanisms that predominantly drive primary resistance—such as a pre-existing immunosuppressive TME and intrinsic oncogenic pathway activation—from those more closely associated with acquired resistance, including compensatory immune checkpoint upregulation, metabolic reprogramming, and epigenetic remodeling, while recognizing that several pathways contribute to both. Where relevant, we consistently distinguish direct evidence demonstrated in ESCC from mechanistic insights extrapolated from other tumor types, explicitly flagging the latter as hypotheses that require validation in esophageal cancer (**Figure 1**).

**Figure 1:**
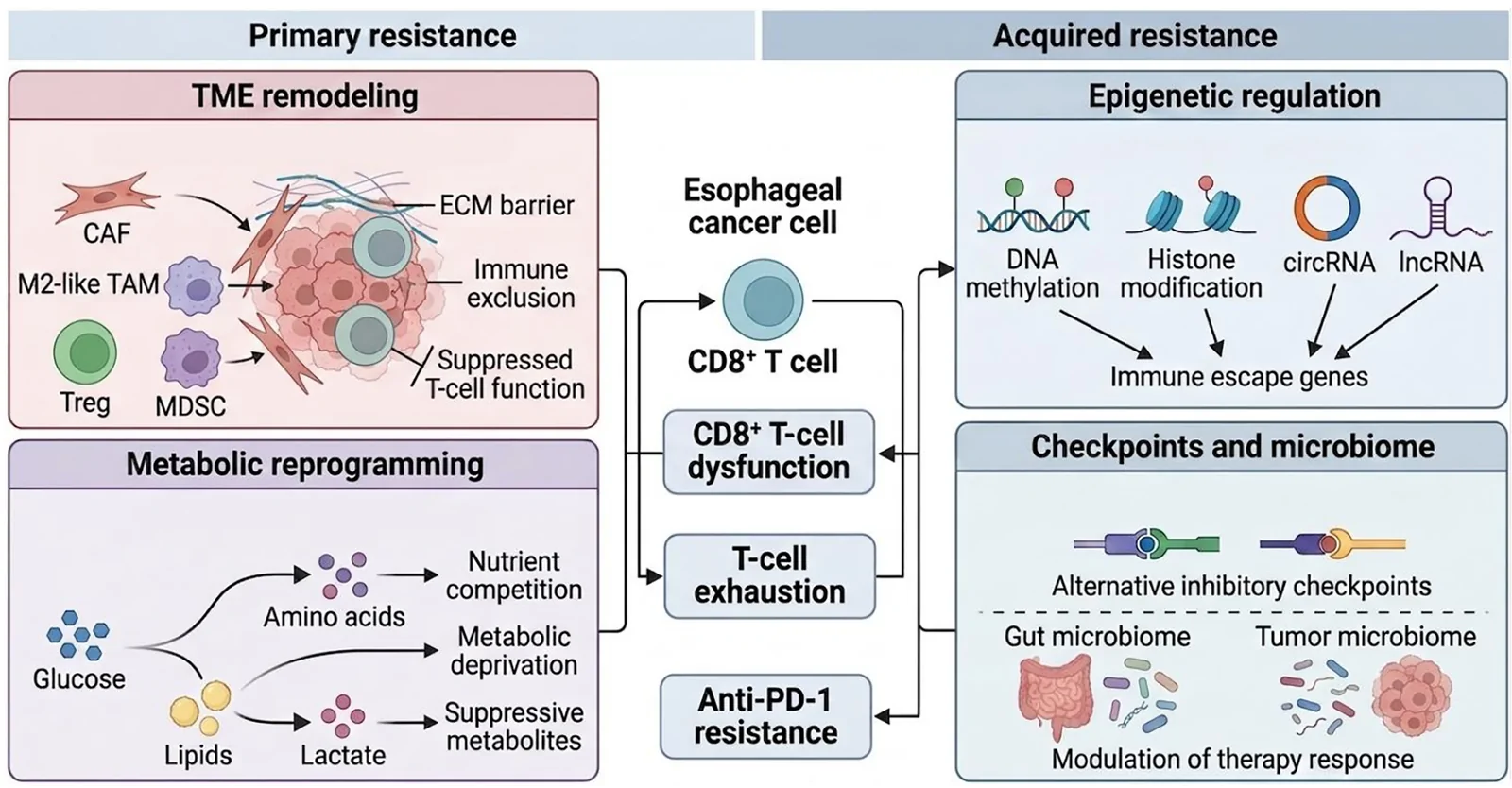
Schematic overview of the major mechanistic axes driving resistance to immune checkpoint inhibitors in esophageal squamous cell carcinoma. The diagram integrates three interconnected axes that cooperatively establish an immunosuppressive tumor microenvironment. Top, CAF axis: CAFs secrete WNT2 and CXCL12, which impair dendritic cell maturation and recruit immunosuppressive MDSCs, leading to reduced priming of T cells. Middle, MDSC/NK axis: MDSCs, often expanded via signals from CD49a^+^ NK cells, produce Arg1 and other suppressive mediators that directly inhibit CD8^+^ T cell function. Together with TGF-β-driven ECM remodeling, this axis promotes the exclusion of CD8^+^ T cells from the tumor core. Bottom, Metabolic & epigenetic reprogramming axis: Metabolic alterations and epigenetic regulators upregulate immune-escape genes and enhance the immunosuppressive function of M2-like macrophages and glycosylated MDSCs. These multilayered mechanisms collectively impair the efficacy of anti-PD-1 therapy. Consistent with the framework introduced in the Introduction, the CAF and MDSC/NK axes predominantly reflect a pre-existing immunosuppressive TME that drives primary resistance, whereas metabolic and epigenetic reprogramming is more closely associated with acquired resistance; several of these mechanisms contribute to both.

## 2 TME remodeling is the main driving factor for immunotherapy resistance

TME represents a dynamic and heterogeneous ecosystem that encompasses not only malignant cells but also various stromal cells, immune cells, extracellular matrix components, and soluble factors. Collectively, these elements influence tumor progression and treatment response (20–22). In EC, the TME is pivotal in facilitating immune escape and fostering resistance to immunotherapy, which is a major cause of treatment failure (23, 24). The immunosuppressive remodeling of the TME establishes a formidable barrier that impedes effective anti-tumor immune responses, consequently diminishing the efficacy of immune checkpoint blockade (25, 26).

### 2.1 Cellular components and their immunosuppressive effects

In the intricate TME of ESCC, various key cellular components play active roles in immunosuppression. Cancer-associated fibroblasts (CAFs) are particularly significant, serving as core coordinators of TME remodeling and immune cell recruitment (27–29). These activated fibroblasts secrete numerous cytokines, chemokines, and extracellular matrix components, thereby fostering the development of an immunosuppressive microenvironment. A critical mechanism by which CAFs enhance drug resistance involves their capacity to recruit and activate other immunosuppressive cell populations, notably tumor-associated macrophages (TAMs) and regulatory T cells (Tregs) (30–32). Epithelial cells activate the NOTCH1 signaling pathway in CAFs through JAG1 ligands, which promotes the secretion of CXCL1 and CXCL8, facilitating the recruitment of immunosuppressive cells and the remodeling of the extracellular matrix (ECM). This interaction creates a “CAF-Epi niche” that impedes T cell infiltration (33). Regarding immune cell interactions, CCL2 and IL-6 secreted by CAFs attract monocytes and induce their differentiation into SPP1+ macrophages. These macrophages, in turn, activate the TNFRSF4 signaling pathway in Tregs by releasing SPP1 protein, establishing a “SPP1 macrophage-Treg” positive feedback loop that inhibits the function of CD8+ T cells (34). Additionally, the WNT2 protein secreted by CAFs can suppress the maturation and antigen presentation capabilities of dendritic cells (DCs), further obstructing T cell activation (35). Furthermore, plasminogen activator inhibitor-1 (PAI-1) secreted by CAFs can trigger the Akt/Erk1/2 pathway (36) in cancer cells, leading to the stimulation of epithelial-mesenchymal transition (EMT) that boosts tumor cell migration capability and resistance to drugs.

TAMs are another key immunosuppressive cell population in the TME of esophageal cancer (37). Studies have demonstrated that the expression levels of characteristic genes, such as SPP1 and APOE, in the highly infiltrating TREM2+ TAM subpopulation within esophageal squamous cell carcinoma tissues are significantly correlated with resistance to anti-PD-1 treatment (38). Specific TAM subsets can induce T cell exhaustion through various mechanisms, including lipid metabolic reprogramming (39). In esophageal squamous cell carcinoma, tumor-cell-secreted cholesterol drives immunosuppressive TAM polarization through a TFEB–PLD3 axis that degrades cytosolic DNA and suppresses TLR9 signaling, thereby impairing T-cell effector functions and promoting a non-responsive or exhausted state. The chemokine CCL22, secreted by TAMs, activates the FAK/AKT/Gli1 signaling pathway in tumor cells. This activation not only induces a stem phenotype in tumor stem cells but also significantly inhibits T cell infiltration (40). Furthermore, TAMs establish a synergistic network with other immunosuppressive cells, including Tregs and myeloid-derived suppressor cells (MDSCs) (37). M2-type TAMs recruit Tregs through the secretion of vascular endothelial growth factor (VEGF) and transforming growth factor-β (TGF-β), while also enhancing the immunosuppressive functions of MDSCs, collectively impeding the anti-tumor effects of CD8+ T cells (41). Nanodelivery systems that target TAMs, such as EA2-PSL-PTX/LUT (42), can diminish the accumulation of these inhibitory cells, thereby reversing the immunosuppressive microenvironment. Concurrently, TAM aggregation in the tumor core creates a formidable physical obstacle that greatly impedes T cell penetration. Single-cell sequencing data revealed a notable association between TREM2+ TAMs and the fibroblast-enriched phenotype in the TME of non-responders to immunotherapy, leading to inadequate immune cycle activation (38, 43). Additionally, research indicates that hypoxia in the TME can trigger the ALCAMhigh phenotype in TAMs, augmenting their immunosuppressive capacity and facilitating immune evasion, a phenomenon yet to be confirmed in esophageal cancer.

Tregs significantly contribute to the immune resistance microenvironment in esophageal cancer, playing a crucial role in the development of immunotherapy resistance. They are excessively present in the esophageal cancer microenvironment, promoting immune evasion through direct suppression of effector T cells, secretion of immunosuppressive factors like interleukin-10 (IL-10) and IL-35, and modulation of the ECM. Analysis of single-cell sequencing data revealed a notable rise in Treg levels within esophageal squamous cell carcinoma tumors, constituting over 30% of CD4^+^ T cells. Moreover, the extent of Treg infiltration positively correlates with unfavorable prognosis and resistance to immunotherapy (44). The differentiation of Tregs is modulated by CAFs. The CAF-secreted protein PI16 promotes the differentiation of naive CD4^+^ T cells into T-BET^+^ Tregs by binding to the DOCK2 receptor, which subsequently inhibits the function of CD8^+^ T cells. Clinical samples have demonstrated that elevated PI16 expression in the interstitium correlates significantly with increased Treg infiltration and a reduced patient survival rate (45). Additionally, Tregs deplete local ATP through the high expression of molecules such as CD39, leading to the generation of adenosine, which inhibits the activity of effector T cells (45, 46). Tregs can sustain a stable immunosuppressive phenotype within the TME via clonal expansion and epigenetic modifications, including the demethylation of the Foxp3 gene (34). In Tregs from non-responders in ESCC, the expression of TNFRSF4 is upregulated, enhancing its inhibitory function through the activation of the NF-κB signaling pathway. Continuous activation of the STAT3 signaling pathway further promotes stable Foxp3 expression, establishing a self-sustaining regulatory loop.

MDSCs represent a heterogeneous population of immature myeloid cells that contribute to immunosuppression and facilitate resistance to immunotherapy within the TME of esophageal cancer through various mechanisms. The expansion of MDSCs significantly promotes tumor progression by directly inhibiting CD8^+^ T cell function, inducing the differentiation of Tregs, and reshaping the immunosuppressive microenvironment. Studies have demonstrated that MDSCs inhibit T cell proliferation and cytotoxicity through the secretion of molecules such as arginase-1 (Arg-1), reactive oxygen species (ROS), IL-10, and TGF-β. Additionally, MDSCs promote angiogenesis and establish an immunosuppressive microenvironment. Specifically, PKN2 (47) enhances the transcription of CPT1B by activating STAT3 phosphorylation, which in turn boosts the fatty acid oxidation (FAO) metabolism of MDSCs, increases the expression of inhibitors like Arg-1 and ROS, and diminishes the anti-tumor efficacy of T cells. Conversely, GPR84 (48) is overexpressed in MDSCs, where it inhibits the lysosomal degradation of PD-L1, thereby sustaining its elevated expression and contributing to resistance against anti-PD-1 therapy. Furthermore, the interaction between MDSCs and other immune cells can contribute to the remodeling of the TME. Exhausted CD49a^+^ NK cells promote MDSC infiltration by secreting granulocyte-macrophage colony-stimulating factor (GM-CSF), a mechanism that may be linked to the poor prognosis observed in early esophageal cancer (49). Additionally, MDSCs can secrete VEGF to facilitate angiogenesis while simultaneously reinforcing the ECM through collagen deposition, thereby impeding immune cell infiltration (47). A recent study by the Institute of Biomedical Experiments in Argentina demonstrated that galectin-1 (GAL1) recognizes β [1,6]-branched N-glycans on the surface of myeloid-derived suppressor cells (MDSCs), binds to the CD18/CD11b/CD177 receptor complex, and activates signaling through the signal transducer and activator of transcription 3 (STAT3) pathway. This activation synergistically upregulates immune checkpoints such as PD-L1 and indoleamine 2,3-dioxygenase 1 (IDO1), as well as the secretion of VEGF, thereby enhancing immunosuppressive and pro-angiogenic functions. In patients resistant to immunotherapy, the downregulation of ST6GAL1, which is responsible for α[2,6]-sialylation, in MDSCs results in the exposure of GAL1 binding sites and further enhances the immunosuppressive phenotype (50).

### 2.2 Physical and biochemical barriers within the TME

In the microenvironment of immunotherapy resistance in ESCC, beyond cellular elements, the physical and biochemical attributes of the TME can impede immune cell infiltration, disrupt immune function, and stimulate the activation of immunosuppressive cells, contributing to immunotherapy failure.

The physical barriers in the TME primarily impede immune cell infiltration by causing ECM fibrosis and vascular irregularities. The accumulation of type I collagen, hyaluronic acid (HA), and fibronectin in the TME leads to the formation of a dense structure, elevating tissue rigidity and impeding the entry of CD8^+^ T cells into the tumor core. In ESCC, the ECM facilitates immune evasion by activating the YAP/TAZ pathway and physically obstructing T cell movement (51). This process may involve collagen-modifying enzymes like LOX, which bolster ECM integrity, raise interstitial hydraulic pressure, and limit the diffusion of therapeutic agents and immune cells (52). Notably, the composition and function of the TME can differ significantly between histological subtypes. For example, EAC is often characterized by a higher degree of immune infiltration but also more pronounced T-cell exhaustion compared to ESCC (26, 51). Furthermore, distinct CAF populations may predominate in each subtype, leading to different therapeutic vulnerabilities.

Abnormal blood vessel formation can elevate interstitial pressure and disrupt blood flow, impeding immune cell transport. Activation of the VEGF pathway by hypoxia-inducible factor-1 α (HIF-1α) stimulates angiogenesis, resulting in the development of an “abnormal vascular network” characterized by heightened vascular permeability and an incomplete basement membrane, thereby impeding immune cell infiltration (53, 54).

The acidic microenvironment characterizes numerous solid tumors, such as esophageal cancer, due to tumor cells’ heightened reliance on aerobic glycolysis (Warburg effect) and consequent lactic acid generation (55). This acidity significantly hampers immune cell activities, particularly CD8^+^ T cells and NK cells, diminishing their cytotoxic functions, cytokine secretion, and replication. Additionally, the low pH levels can modify the expression of immune checkpoints and other suppressive molecules on both tumor and immune cells, thereby advancing immune evasion (56).

Hypoxia is a common characteristic of the TME in esophageal cancer, resulting from rapid tumor growth outpacing vascular development. Hypoxic conditions trigger the activation of hypoxia-inducible factors (HIFs) (57, 58) which subsequently control the expression of various genes related to angiogenesis, metabolism, and immunosuppression. Hypoxia induces the autophagy-mediated reduction of major histocompatibility complex (MHC-I) molecules by activating the PERK pathway of the unfolded protein response, diminishing tumor antigen presentation and avoiding immune detection. Additionally, hypoxia elevates HIF-1α/HIF-2α levels, inducing metabolic reprogramming and increasing metabolites like lactic acid and adenosine. This environment suppresses T cell function, fosters M2-type macrophage polarization, and expands Treg populations (59). Concurrently, the hypoxic conditions enhance PD-1 expression in CD8^+^ T cells, leading to functional exhaustion characterized by decreased secretion of granzyme B and IFN-γ (60).

Moreover, the nutrient-depleted microenvironment in the TME, marked by the rivalry for critical nutrients like glucose and amino acids, exacerbates the immune cells’ functionality. Tumor cells, which have elevated metabolic requirements, vie with immune cells for these vital resources, resulting in nutrient scarcities and functional deficiencies in anti-tumor immune cells. This metabolic rivalry plays a crucial role in drug resistance and will be extensively discussed in upcoming sections.

The physical and biochemical barriers work together to establish an immune-privileged microenvironment shielding tumor cells from immune monitoring and treatment. The ever-changing TME restructuring (61) poses a significant obstacle in esophageal cancer immunotherapy. While many of these TME principles are extrapolated from broader solid tumor research, direct evidence from single-cell studies in EC has confirmed the crucial role of specific components, such as immunosuppressive fibroblast subsets and myeloid cells, in driving resistance (24, 34). Grasping the intricate interplay among tumor cells, stromal cells, and immune cells in this complex system is vital for devising successful approaches to combat drug resistance. Building on this direct link where metabolites modulate the epigenome, the following section will explore the broader landscape of epigenetic dysregulation and non-coding RNAs as fundamental drivers of immunotherapy resistance. Directing interventions toward TME elements or standardizing TME’s physical characteristics is anticipated to boost the effectiveness of current immunotherapy and enhance the outlook for esophageal cancer patients (**Figure 2**).

**Figure 2:**
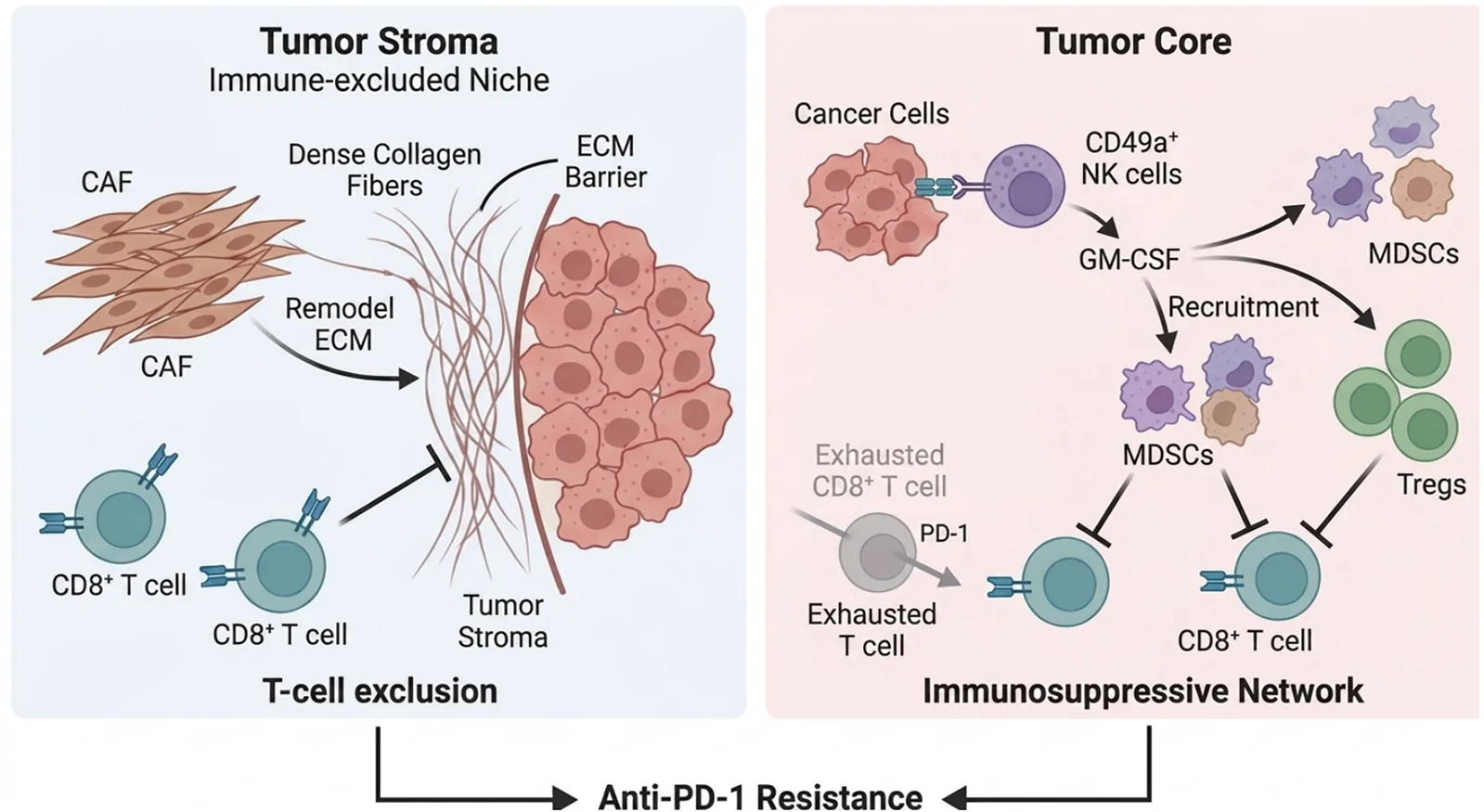
Dual immunosuppressive mechanisms driving anti-PD-1 resistance in ESCC. The schematic illustrates two interconnected axes within the tumor stroma that collectively establish an immune-excluded niche and promote T-cell dysfunction. Left (physical barrier axis): CAFs remodel the ECM by depositing dense collagen fibers, forming a physical ECM barrier that impedes CD8^+^ T-cell infiltration into the tumor core, leading to T-cell exclusion. Right (cellular immunosuppression axis): ESCC cells and CD49a^+^ NK cells secrete granulocyte-macrophage colony-stimulating factor (GM-CSF), which recruits MDSCs and Tregs into the tumor microenvironment. These immunosuppressive populations directly suppress CD8^+^ T cells, driving their exhaustion (exhausted CD8^+^ T cells, PD-1^+^). The combined effect of physical exclusion and functional impairment of CD8^+^ T cells results in resistance to anti-PD-1 immunotherapy.

## 3 Metabolic reprogramming is a key hub connecting the interaction between tumors and immune cells

The metabolic diversity of the TME in esophageal cancer plays a crucial role in determining the effectiveness of immunotherapy. Tumor cells alter their metabolic pathways utilizing the Warburg effect, amino acid consumption, and lipid accumulation, leading to the development of a “metabolic desert” TME characterized by nutrient scarcity, hypoxia, acidosis, and the buildup of immunosuppressive factors. Furthermore, the dysregulated metabolism in the TME acts as a central node, intricately linking tumor cells to immune cells and significantly impacting the response to immunotherapy in esophageal cancer.

### 3.1 Glucose metabolism and lactic acid accumulation

In ESCC, tumor cells exhibit the Warburg effect, leading to significant lactic acid accumulation in the TME (55). Lactic acid, a glycolysis by-product, contributes to the TME’s acidic pH, directly hindering T cell function and fostering immunosuppression. Moreover, elevated levels of hexokinase 2 (HK2) and lactate dehydrogenase (LDH) in esophageal cancer can hinder T cell function by inducing glycolysis and lactic acid buildup, resulting in energy insufficiency (62). The identification of histone lactylation, a post-translational modification (PTM) occurring at histone lysine sites (63), underscores the significant influence of lactic acid on epigenetic regulation and gene expression within the TME. This modification establishes a direct connection between tumor lactate metabolism and epigenetic alterations that can impact immune infiltration and energy metabolism, thereby exacerbating immunosuppression. In solid tumors, lactylation of METTL16 (64) has been shown to promote cuproptosis in gastric cancer, suggesting that non-histone lactylation plays a broader role in tumor biology and potential therapeutic strategies. Although this specific example pertains to gastric cancer, the principle by which lactate-mediated PTMs influence cellular processes is highly relevant to esophageal cancer (65).

### 3.2 Amino acid metabolism disorder

Amino acid metabolic disorders, alongside glucose, significantly influence the immunosuppressive TME in ESCC (66). Research indicates that ESCC macrophages enhance Arg1 expression via the ISCU-p53 axis, which contributes to T cell exhaustion (67). The uptake of cystine through SLC7A11 depletes cysteine in the surroundings, hindering T cell glutathione (GSH) production, thereby promoting oxidative stress and ferroptosis. Furthermore, the upregulation of indoleamine 2, 3-dioxygenase (IDO) leads to tryptophan depletion and kynurenine accumulation, which suppresses T cell function and fosters an immunosuppressive microenvironment (68). The consumption of arginine represents another critical mechanism of immunosuppression (69). As an essential amino acid for T cell activation, arginine depletion impairs the proliferation and cytotoxic capabilities of CD8+ T cells. Although Arg1/2 is downregulated in esophageal cancer, other immunosuppressive cells within the TME, such as M2-type TAMs and MDSCs, may further deplete arginine through elevated expression of Arg2, resulting in localized immunosuppression. Additionally, Arg2 is associated with hypoxia-inducible factor (HIF-2α). Under hypoxic conditions, the upregulation of Arg2 can prompt macrophages to adopt tumor-promoting M2 phenotypes, thereby inhibiting anti-tumor immunity.

Furthermore, the accumulation of adenosine (70) within the TME, typically resulting from the activity of exonucleases such as CD39 and CD73 on tumor and immunosuppressive immune cells, directly inhibits T cell activation and facilitates immune escape. Adenosine transmits signals through the A2A receptor on immune cells, which inhibits their effector functions and promotes the differentiation of immunosuppressive cell types.

### 3.3 Disorder of lipid metabolism and choline metabolism

Abnormal lipid metabolism (71) significantly contributes to immune escape and resistance to immunotherapy in ESCC. The uptake of fatty acids by C1q^+^ TAMs (72) has been shown to promote an immunosuppressive phenotype and induce T cell exhaustion (39). In esophageal squamous cell carcinoma, tumor-cell-secreted cholesterol drives this immunosuppressive TAM polarization through a TFEB–PLD3 axis that degrades cytosolic DNA and suppresses TLR9 signaling. These TAMs utilize fatty acids as an energy source, which may further influence their polarization and function, thereby enhancing the immunosuppressive TME. Cholesterol metabolism exhibits a dual nature. The deficiency of acetyl-CoA acetyltransferase 1 (ACAT1) can elevate cholesterol levels in T cell membranes and improve the formation of immune synapses. Conversely, excessive cholesterol induces endoplasmic reticulum stress, which promotes T cell exhaustion (56). Dysregulated choline metabolism (73) represents another emerging area of research. Mthfd1-mediated TAM polarization contributes to angiogenesis and immune escape by reshaping the immune microenvironment. Choline metabolism is essential for membrane synthesis and signal transduction. Its dysregulation can impact cell proliferation and differentiation, thereby influencing the overall immune landscape of tumors, a topic that requires further investigation in esophageal cancer.

### 3.4 Metabolism-epigenetic cross-regulation

The intricate relationship between metabolism and epigenetics is crucial for understanding the regulation of drug resistance in immunotherapy. For example, the Warburg effect, common in EC, leads to high levels of lactate in the TME. This lactate is not merely a waste product; it serves as a substrate for histone lactylation, a novel epigenetic modification that can directly suppress the expression of genes crucial for T-cell activation and effector function, thus driving a resistance phenotype (74). In esophageal cancer, a NIPAL1–HCK–p-LDHA–lactate–p300–H3K18la positive-feedback loop sustains this histone lactylation and impairs CD8+ T-cell function, and its inhibition restores anti-tumor immunity and sensitizes tumors to anti-PD-1 therapy. Similarly, alterations in the TCA cycle can impact the availability of acetyl-coenzyme A (75), the essential donor for histone acetylation, leading to widespread changes in gene expression that favor immune evasion. A notable example of this connection is phosphoenolpyruvate carboxykinase 1 (PCK1) (76) a pivotal enzyme in gluconeogenesis. PCK1 influences the TME not only through its established role in glucose metabolism but also via non-classical functions in signaling pathways that may involve epigenetic modifications or impact the functions of immune cells and the behavior of tumor cells. This underscores a complex regulatory network in which metabolic changes can directly modify the epigenetic landscape, resulting in alterations in gene expression that facilitate immune evasion and drug resistance. The notion of metabolites as direct regulators of epigenetic modifications further reinforces this critical connection.

Taken together, these observations illustrate that the TME, metabolic reprogramming, and epigenetic regulation do not operate as isolated layers but as an integrated, self-reinforcing system. Hypoxia—a hallmark of the ESCC TME—simultaneously rewires glucose and lipid metabolism, impairs antigen presentation, and stabilizes transcription factors that drive epigenetic remodeling, while tumor-derived lactate and other metabolites feed back onto the epigenome through histone lactylation and acetylation. These cross-layer interactions converge on a common output of impaired T-cell effector function and elevated immune-checkpoint expression, underscoring that rational combination strategies must target this system as a whole rather than any single layer in isolation.

### 3.5 Metabolic-mediated resistance to cell death and immune editing

Metabolic reprogramming equips tumor cells with resistance to various forms of cell death while influencing the immune editing landscape. As previously noted, cuproptosis, a novel form of cell death, is intricately regulated by lactic acid metabolism in ESCC. Research conducted by Sun Yat-sen University demonstrated that the L-lactate-NUDT21 lactoylation-FDX1 inhibitory axis allows tumor cells to evade death induced by copper ion carriers, such as Elesclomol. Moreover, elevated levels of NUDT21 lactylation correlate with poor prognosis in patients (77). The ISCU-p53 axis in macrophages facilitates M2 polarization by mitigating the transcriptional repression of SLC7A11, a key negative regulator of ferroptosis, and Arg1 by p53. Inhibition of ISCU expression can enhance the sensitivity of macrophages to ferroptosis, augment the cytotoxicity of CD8^+^ T cells, and improve the efficacy of anti-PD-1 therapy (67). Furthermore, multi-omics analysis from the MEMORI trial (51) revealed that during neoadjuvant therapy for esophageal adenocarcinoma, 46% of non-responders (NRP) exhibited loss of heterozygosity (LOH) for HLA, a significantly higher rate than that observed in responders (REP, 13%). This hereditary defect in antigen presentation, coupled with the upregulation of PD-L1 transcription, establishes a “double escape” mechanism. Additionally, metabolic stress, such as hypoxia, further enhances immune editing by inducing downregulation of HLA-I molecule expression (78).

In conclusion, metabolic reprogramming serves as a central hub within the intricate network of drug resistance in esophageal cancer immunotherapy. Tumor cells and associated stromal cells actively create an immunosuppressive microenvironment that is rich in nutrients and metabolites, thereby disabling anti-tumor immune cells. Targeting these metabolic vulnerabilities—through the restoration of nutritional supplies to immune cells, the inhibition of immunosuppressive metabolic pathways, or the disruption of metabolism-epigenetic cross-regulation—presents promising strategies for overcoming drug resistance and enhancing the efficacy of ICIs in esophageal cancer (**Figure 3**).

**Figure 3:**
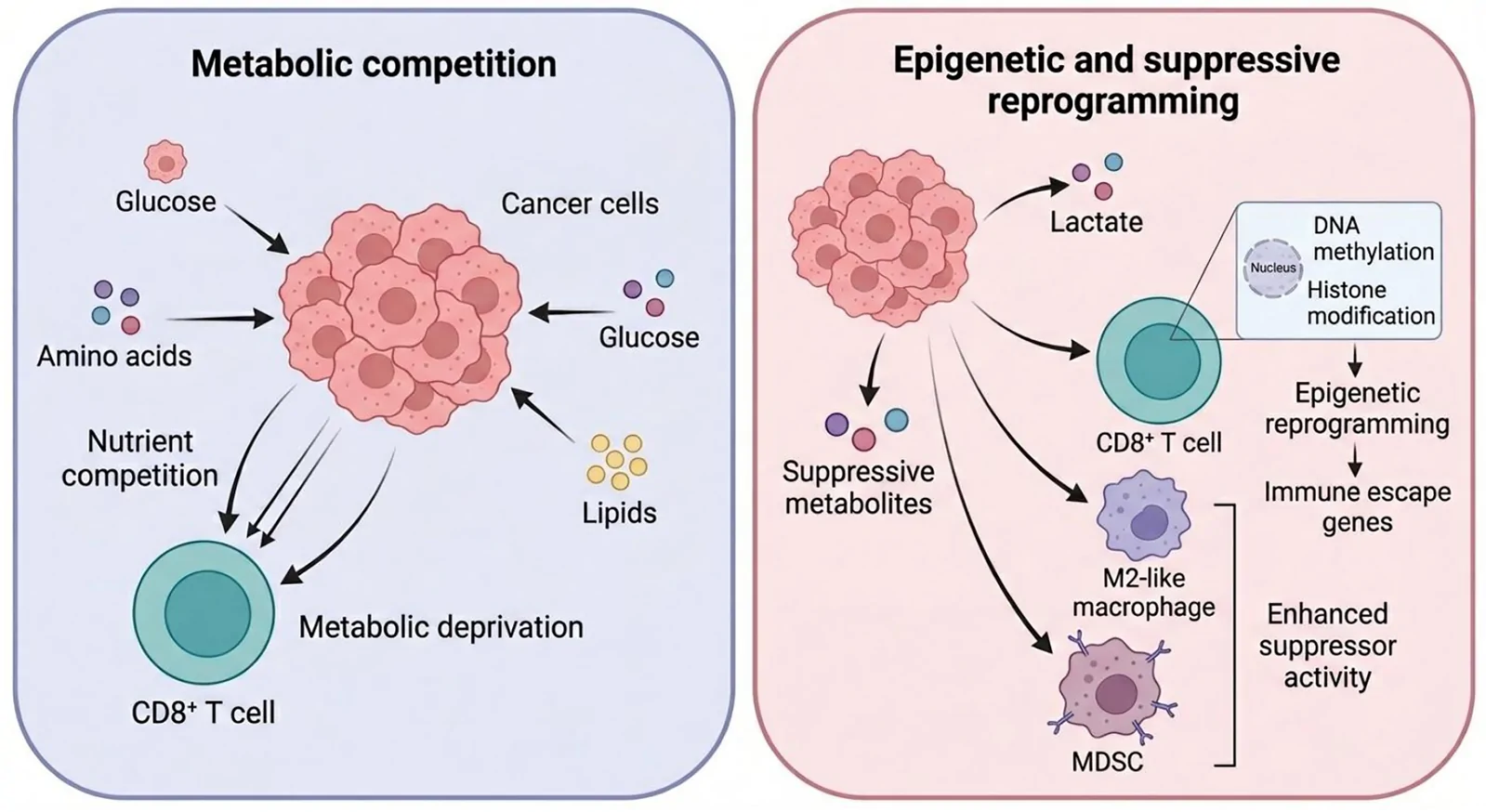
Metabolic reprogramming orchestrates CD8^+^ T-cell dysfunction and immunosuppression in ESCC. This schematic illustrates two interconnected mechanisms by which metabolic alterations in the TME promote immune evasion. Left panel (Metabolic competition): ESCC cells avidly consume glucose, amino acids, and lipids, creating a nutrient-depleted environment that restricts metabolite availability for CD8^+^ T cells, leading to CD8^+^ T cell metabolic deprivation and impaired effector function. Right panel (Suppressive metabolites and epigenetic reprogramming): Tumor-derived lactate and other suppressive metabolites act on M2-like TAMs and MDSCs, enhancing their immunosuppressive activity. Concurrently, these metabolites induce epigenetic reprogramming, including DNA methylation and histone modifications, which upregulate the expression of immune-escape genes. Collectively, these processes contribute to an immunosuppressive metabolic niche that fosters CD8^+^ T-cell dysfunction and resistance to anti-PD-1 immunotherapy.

## 4 Epigenetic regulation and non-coding RNA enhancement of drug resistance networks

In addition to gene mutations, epigenetic alterations and the dysregulation of non-coding RNAs are critical regulatory factors that influence gene expression without changing the underlying DNA sequence. These dynamic modifications significantly contribute to the TME, modulate immune cell function, and ultimately enhance the drug resistance mechanisms of ESCC to immunotherapy (79). Furthermore, the accessibility of epigenetic modifications offers a promising avenue for therapeutic intervention, potentially rendering drug-resistant tumors more susceptible to immune checkpoint blockade.

DNA methylation predominantly occurs at CpG dinucleotides and serves as a crucial epigenetic marker. Methylation of CpG sites within promoter regions typically results in gene silencing. A multi-omics study (80) classified ESCC into four molecular subtypes for the first time. Among these, the cell cycle pathway activation type (CCA) and the NRF2 pathway activation type (NRFA) were identified as E-CIMP+ and were significantly associated with lymph node metastasis and advanced clinical stage. In these patients, hypermethylation leads to the silencing of key genes involved in the antigen presentation mechanism (APM), such as B2M, thereby weakening the tumors’ ability to present neoantigens and hindering CD8+ T cells from effectively recognizing tumor cells. A critical mechanism by which DNA methylation promotes immune escape is the silencing of MHC class I molecule expression (81, 82). MHC class I molecules are essential for presenting tumor-associated antigens to CD8^+^ cytotoxic T lymphocytes (CTLs), enabling these cells to recognize and eliminate malignant cells. When MHC class I expression is downregulated or lost due to hypermethylation of the promoter, tumor cells become “invisible” to the immune system, thus evading T-cell-mediated killing, even in the presence of immune checkpoint blockade. This epigenetic silencing of the antigen-presenting pathway represents a significant mechanism underlying both primary and acquired resistance to ICIs in various cancers, including esophageal cancer. In contrast, promoter hypomethylation represents an independent regulatory mechanism that may activate oncogenes and immune-escape-related genes such as PD-L1. Research indicates that hypomethylation of the PD-L1 promoter region correlates with its overexpression in ESCC cells (83), thereby contributing to the development of immunotherapy resistance.

Histone modification refers to covalent post-translational modifications (PTMs) of histone proteins, including methylation, phosphorylation, acetylation, and ubiquitination, among others. These modifications influence gene expression by modulating the interaction between histones and DNA or by facilitating binding to effector proteins (63, 65, 84). In a panel of ESCC cell lines (KYSE-450, KYSE-150, KYSE-510, and KYSE-30), HDAC activity is markedly elevated relative to the normal esophageal epithelial cell line Het-1A (85), although whether this elevation represents a general feature of ESCC remains to be established. Research indicates that histone deacetylase inhibitors (HDACI) can directly modulate gene expression and signal transduction in mouse CD8+ T cells and CD4+ T cells. By enhancing the expression of effector cytokines such as TNF-α and IFN-γ, as well as increasing the cytotoxicity of CD8+ T cells, these inhibitors promote T cell effector functions. However, depending on the specific inhibitor, dose, treatment duration, and experimental model, they may also contribute to T cell exhaustion and the enrichment of immunosuppressive myeloid cells (86). Moreover, HDACI can regulate EMT, a process linked to drug resistance and immune evasion (87, 88). The effect of HDAC inhibition on EMT appears to be context-dependent, varying with the specific inhibitor and cellular context. Together, these findings suggest that histone modifications may influence the development of drug resistance in immunotherapy, although the net effect of HDAC inhibition on anti-tumor immunity requires further clarification. EZH2, a histone methyltransferase, catalyzes the trimethylation of histone H3 at Lys 27 (H3K27me3), thereby promoting transcriptional silencing and influencing differentiation. It is a significant factor contributing to resistance against immune checkpoint blockade (ICB) (89). EZH2 is overexpressed in various cancers, including esophageal cancer, and is involved in promoting cell survival, proliferation, EMT, invasion, and drug resistance (90). Inhibiting EZH2 can modulate the immune microenvironment and enhance anti-tumor immunity, positioning EZH2 inhibitors as a promising strategy to overcome drug resistance in immunotherapy (91, 92).

Non-coding RNAs (ncRNAs), which include microRNAs (miRNAs), long non-coding RNAs (lncRNAs), and circular RNAs (circRNAs), serve as crucial regulators of gene expression. These ncRNAs regulate gene expression through epigenetic, transcriptional, and post-transcriptional mechanisms, thereby establishing complex interaction networks. These ncRNAs play a vital role in the onset, progression, immune evasion, and drug resistance of various tumors, including esophageal cancer (93–96).

CircRNAs have emerged as pivotal components of the drug-resistant network, attributed to their stable circular structure and high tissue specificity. Immune evasion may occur via miRNA sponging or through direct regulation of immune checkpoints, such as PD-L1 (97). In patients with ESCC, CircNF1 is significantly upregulated in both tissues and serum, correlating closely with responses to anti-PD-L1 immunotherapy. Research indicates that CircNF1 regulates PD-L1 through two mechanisms: transcriptionally via the JAK-STAT3 pathway and post-translationally through the interaction between ANXA1 and USP7. This regulation promotes the progression of ESCC and facilitates immune escape (98).

LncRNAs represent a diverse class of non-coding RNAs, characterized by lengths exceeding 200 nucleotides, and are involved in various cellular processes, including chromatin remodeling, gene transcription, and post-transcriptional regulation (99). Previous studies have suggested that lncRNAs may be associated with resistance to chemotherapy (100) and radiotherapy (101) in esophageal cancer. A recent investigation (68) revealed that lncRNA RIME (LINC02096) in ESCC can inhibit the ubiquitination of mixed lineage leukemia protein-1 (MLL1) through direct binding. This interaction enhances MLL1’s stability, which subsequently increases the enrichment of H3K4me3 in the promoter regions of PD-L1 and IDO-1. As a result, the expression of the immune checkpoint PD-L1 and the immunoregulatory enzyme IDO1 is upregulated, contributing to CD8+ T cell dysfunction and facilitating tumor immune escape.

In conclusion, epigenetic modifications and non-coding RNAs constitute a complex regulatory network that profoundly affects the response of esophageal cancer to immunotherapy. By modulating antigen presentation, T-cell function, and the expression of immune checkpoints, these mechanisms play critical roles in both primary and acquired drug resistance. Furthermore, they present promising targets for improving the efficacy of ICIs and for overcoming drug resistance in esophageal cancer.

## 5 Other mechanisms

### 5.1 Compensatory immune checkpoints are upregulated

Following the successful inhibition of the PD-1/PD-L1 pathway, the immune system may remain constrained by other inhibitory pathways, which are compensatorily activated to establish a complex immunosuppressive network (102, 103). Cytotoxic T lymphocyte-associated protein 4 (CTLA-4) (104, 105) competes with the co-stimulatory molecule CD28 on T cell surfaces for binding to the B7 molecule (CD80/CD86) found on antigen-presenting cells, such as dendritic cells. This competition inhibits the initial activation and proliferation of T cells, thereby diminishing the immune response. The effectiveness of anti-CTLA-4 antibodies, such as ipilimumab, as monotherapy in esophageal cancer is limited. Currently, these antibodies are more frequently employed in combination with anti-PD-1/PD-L1 antibodies (106, 107) to enhance anti-tumor immunity through the dual blockade of immune checkpoints, demonstrating synergistic effects in several clinical trials. T-cell immunoglobulin and ITIM domain proteins (TIGIT) (108), part of the immunoglobulin superfamily, exhibit structural characteristics common to various immunomodulatory proteins. These proteins are expressed in activated CD8^+^ T cells, CD4^+^ T cells, NK cells, and Tregs. The compensatory upregulation of TIGIT on T cells following PD-1/PD-L1 blockade may contribute to acquired drug resistance, allowing tumor cells to persist in evading immune surveillance. TIGIT inhibits T cell activation and promotes an immunosuppressive phenotype by competing with CD226 for binding to CD155. Furthermore, Tregs that express high levels of TIGIT exert more potent immunosuppressive effects, while NK cells are more susceptible to exhaustion, collectively facilitating tumor immune escape. Early clinical trials have shown that the combination of TIGIT inhibitors and PD-L1 inhibitors exhibits promising anti-tumor activity in advanced esophageal cancer (109). Lymphocyte activating gene 3 (LAG-3) (110) is an inhibitory immune checkpoint protein. Similar to PD-1 and CTLA-4, LAG-3 is not expressed on naive T cells. Upon antigen stimulation, it can be induced on CD4+ and CD8+ T cells, where it participates in transmitting inhibitory signals that diminish T cell effector function and promote T cell exhaustion (111). A clinical trial evaluating the combination of LAG-3 inhibitors with PD-1 inhibitors in neoadjuvant therapy for patients with resectable gastric and esophageal cancer demonstrated favorable therapeutic effects and a reliable safety profile (112). Additionally, PD-L1/TIGIT and PD-L1/LAG-3 have emerged as optimal combinations for predicting the prognosis of esophageal cancer and serve as effective targets for enhancing the efficacy of immunotherapy in this context (113, 114). T-cell immunoglobulin mucin-3 (115, 116) is predominantly expressed on the surface of activated T cells, NK cells, dendritic cells, and macrophages, with particularly high expression in exhausted T cells. Previous studies have established that TIM-3 not only directly inhibits T cell function but also fosters the development of a tumor immunosuppressive microenvironment by modulating the activities of other immune cells, including MDSCs and Tregs (49, 117). PILRα, a novel immunomodulatory molecule, is markedly overexpressed in triple-negative breast cancer (TNBC), cholangiocarcinoma (CHOL), EC, and glioblastoma (GBM). Its expression positively correlates with immune checkpoint molecules such as PD-1, CTLA-4, and TIM-3, indicating a potential synergistic effect. Furthermore, PILRα demonstrates a significant negative correlation with the infiltration of CD8+ and CD4+ T cells, which is indicative of a distinct immune rejection phenotype (118). B7-H4 serves as another critical molecule involved in immune evasion in esophageal cancer. Research has shown that esophageal cancer cells infected with *Porphyromonas gingivalis (Pg)* exhibit elevated levels of B7-H4, facilitating tumor cell evasion of host immune surveillance. Additionally, the histone demethylase KDM5B has been identified as functioning in concert with immune checkpoints to collaboratively establish an immunosuppressive microenvironment, thereby conferring survival advantages to tumor cells (119).

### 5.2 The role of the microbiome in immunotherapy drug resistance

Beyond the intrinsic mechanisms of tumors and the factors within the TME, the microbiome has emerged as a crucial regulator of anti-tumor immunity and the response to immunotherapy (120, 121). Research involving patients with esophageal cancer has revealed significant differences in microbiome diversity and composition between responders and non-responders (122). Patients with ESCC who responded to neoadjuvant chemotherapy combined with immunotherapy (NACI) exhibited a higher abundance of *Streptococcus* spp. in their tumors, which correlated with an extended disease-free survival period. Furthermore, another study indicates that the presence of intestinal *Bifidobacterium* (123) is linked to a decrease in FOXP3+ regulatory T cells in the tumor tissues of ESCC patients, suggesting its potential role in overcoming drug resistance by enhancing the anti-tumor immune response. Furthermore, recent studies reinforce this connection; for instance, specific microbial signatures within the tumor have been correlated with favorable responses to neoadjuvant ICIs in ESCC patients (124). On a mechanistic level, certain microbial metabolites have been shown to directly modulate tumor cell sensitivity to ICIs or influence immune cell function within the TME, representing novel therapeutic targets (125). Additionally, bacteria such as *Pg* and *Fusobacterium nucleatum (Fn)* can promote cancer development by producing inflammatory factors or directly invading the esophageal mucosa, significantly impacting the efficacy of treatments including chemotherapy, immunotherapy, and targeted therapy (126–128).

*Pg* is a Gram-negative anaerobic bacterium that has been closely linked to esophageal cancer. Research indicates that the detection rate of its colonization in esophageal cancer cases reaches 42%, and this infection is statistically correlated with the clinicopathological features of the tumor as well as the 5-year survival rate of patients. The bacterium can stimulate the release of pro-inflammatory factors, such as IL-6 and IL-8, through the secretion of gingipains (129). Furthermore, it has been shown to promote the high expression of the immune checkpoint molecule B7-H4 and the histone demethylase KDM5B in esophageal cancer cells, thereby undermining the host immune response (119).

*Fn* is a prevalent Gram-negative anaerobic bacterium that is frequently found in elevated levels in upper gastrointestinal malignancies, including esophageal and gastric cancers. Prior studies have indicated that *Fn* can facilitate the development and metastasis of esophageal cancer while also contributing to chemotherapy resistance (130–132). One investigation revealed (133) that among patients with ESCC receiving PD-1 inhibitors, the levels of *Fn* DNA enrichment and serum *Fn* antibodies in the non-responder (NR) group were significantly greater than those in the responder (R) group. Animal studies have demonstrated that *Fn* infection diminishes the efficacy of PD-L1 treatment and may even prevent the inhibition of tumor metastasis in *Fn*-infected mice. Mechanistic studies have shown that *Fn* can infiltrate esophageal cancer cells, allowing it to survive and proliferate. Its virulence factor, *Fn-Dps*, binds to the activating transcription factor 3 (ATF3) within the PD-L1 gene promoter, leading to the transcriptional upregulation of PD-L1 expression. Additionally, *Fn* can suppress T cell proliferation and the secretion of cytokines such as IFN-γ and TNF-α, thereby promoting tumor progression and resistance to immunotherapy. In esophageal cancer, co-infection with *Pg* and Fusobacterium nucleatum has been shown to upregulate PD-L1 in tumor tissues through NF-κB signaling (133). *Fn* has also been shown to upregulate PD-L1, either by activating the TLR4/NF-κB pathway or by binding the virulence factor *Fn-Dps* to ATF3 (133). Consequently, further studies have demonstrated that *Pg* and *Fn* can co-aggregate to form biofilms, thereby enhancing each other’s virulence and potentially further upregulating PD-L1 through the activation of overlapping pathways (134).

While direct evidence linking the microbiome to immunotherapy resistance in esophageal cancer is still developing, its known tumor-promoting and immunosuppressive effects in other gastrointestinal cancers indicate a similar potential in esophageal cancer. Modulating the intestinal microbiome through fecal microbiota transplantation, probiotics, or antibiotics presents a novel approach to enhancing the efficacy of immunotherapy and addressing drug resistance.

The mechanisms detailed in this review contribute differentially to primary and acquired resistance. For instance, a pre-existing non-inflamed or “cold” TME, characterized by a lack of T-cell infiltration and a high abundance of immunosuppressive cells like CAFs and MDSCs, is a major driver of primary resistance. Similarly, intrinsic tumor factors such as oncogenic pathway activation are often associated with an initial lack of response. In contrast, acquired resistance frequently arises from adaptive changes during therapy, such as the upregulation of compensatory immune checkpoints (e.g.,VISTA, TIM-3), metabolic reprogramming of tumor cells to evade immune pressure, or epigenetic modifications that lead to stable suppression of antigen presentation machinery.

## 6 Overcoming drug resistance: joint strategies and future directions

The intricate and multi-faceted nature of drug resistance in esophageal cancer immunotherapy has ushered in an era characterized by combination therapies. In light of the challenges posed by drug resistance in both single-agent immunotherapy and chemotherapy-immunotherapy combinations, innovative strategies grounded in the analysis of drug resistance mechanisms have emerged in recent years. These strategies have achieved significant breakthroughs, spanning from clinical applications to fundamental research. Focused on “reversing the immunosuppressive microenvironment, reshaping immune response activity, and obstructing pathways of drug resistance,” these combination approaches, when paired with precise molecular typing and technological advancements, hold promise for overcoming the bottlenecks associated with drug resistance. Concurrently, optimizing molecular typing, developing novel biomarkers, and integrating interdisciplinary technologies represent key avenues for future research.

### 6.1 The verified core combination therapy strategy

Combination therapy utilizing ICIs is being investigated as a promising strategy for immune re-challenges. For example, anti-angiogenic agents enhance the efficacy of immunotherapy by ameliorating the hypoxic conditions within the TME and decreasing the infiltration of immunosuppressive cells. CAP 02 (135) re-evaluated the treatment approach. Camrelizumab, in conjunction with Apatinib, was administered to patients with previously unsuccessful immunotherapy and combination therapy, as well as those with unresectable locally advanced, locally recurrent, or metastatic ESCC. The ORR was 10.2%, the disease control rate (DCR) was 69.4%, the median progression-free survival (mPFS) was 4.6 months, and the median overall survival (mOS) reached 7.5 months. The most favorable therapeutic outcomes were observed in patients exhibiting positive PD-L1 expression and an immune response to first-line treatment, with mPFS reaching 5.7 months and mOS extending to 9.6 months. The safety profile is reliable, establishing this regimen as one of the standard treatment options following drug resistance. Mechanistically, anti-angiogenic therapy disrupts glutamate metabolic crosstalk in SLC1A3-highly expressed tumor cells and myofibroblasts, reduces extracellular matrix deposition, and alleviates the immune barrier. Professor Wang Feng’s team further demonstrated that patients with the “TP53+/FAT1+NOTCH3-” (136) gene signature derived greater benefit from the “target-free” regimen of Anlotinib combined with Benmelstobart, thereby providing a molecular marker for precise combination therapy. Specifically, the TP53+/FAT1+/NOTCH3- signature identifies a subset of ESCC patients with intact antigen presentation machinery and low baseline Treg infiltration, who are more likely to achieve an objective response when receiving this chemo-free regimen.

Antibody-drug conjugates (ADCs) that target immune resistance-related markers, when combined with immunotherapy, have emerged as a promising avenue in recent years. BL-B01D1 (137), the first bispecific ADC targeting EGFR/HER3, was evaluated in a Phase Ib trial involving previously treated patients with metastatic ESCC. Patient selection in this study was based on baseline EGFR/HER3 expression levels (H-score ≥ 150 by immunohistochemistry), as preclinical data indicated that dual targeting is most effective when both receptors are expressed at moderate-to-high levels. This trial achieved an ORR of 29.3% and a mOS of 11.5 months, significantly surpassing traditional second-line therapies. The combination of the anti-PD-1 antibody (SCT-I10A) with the anti-EGFR antibody (SCT200) also exhibited anti-tumor activity in patients who had previously failed immunotherapy, yielding an ORR of 17.24% (138). Furthermore, B7H3 ADC (139) and Nectin-4 ADC (140, 141) have shown manageable safety profiles and potential efficacy in early clinical trials. By targeting specific antigens on the surface of tumor cells, these ADCs induce tumor cell death while releasing antigens that enhance the immune response, thereby creating a synergistic effect against drug resistance. Other combination strategies, including immunotherapy paired with oncolytic viruses (142, 143), dual immunotherapy, and immunotherapy combined with localized radiotherapy, have also demonstrated significant results in the early stages of clinical trials (**Table 1**).

**Table 1:** Selected clinical trials and registered studies investigating strategies to overcome immunotherapy resistance in esophageal cancer.

Trial	Phase	Treatment	Type	Primary outcome measures
NCT06304974 (137, 159)	III	BL-B01D1	ADC	PFS; OS
CTR20234222 (160)	III	KC1036	Targeted therapy	OS
CTR20244846 (161)	II	HLX43	ADC	ORR; PFS
NCT07203898 (162)	I	IPM514	Tumor vaccine	AEs; PK parameter
NCT06697665 (163)	I	LM103	TIL	AEs
CTR20243483 (164)	III	Simmitinib	Targeted therapy	OS
CTR20243305 (165)	I/II	SCTB41	TsAbs	AEs; DLT; RP2D; ORR
CTR20230518 (166)	Ib	FC084CSA	Targeted therapy	DLT; MTD; RP2D; AEs; ECG
CTR20251166 (167)	I	HSK41959	Targeted therapy	AEs
CTR20232756 (168)	I	LS-HB	Photosensitizer	AEs
CTR20233925 (169)	I	UCMYM802	CAR-T	DLT; AEs
CTR20233999 (170)	I	LM103	TIL	DLT; AEs
CTR20222514 (171)	I	LTC004	Cytokines	DLT; AEs; MTD;PR2D; ORR
CTR20212713 (172)	Ia	QLF31907	BsAbs	DLT; MTD; RP2D
CTR20243795 (173)	Ia	GTA182	Targeted therapy	DLT; AEs
CTR20231668 (174)	I	hV01	Oncolytic Viruses	DLT; AEs
NCT06681285 (175)	II	Cadonilimab; Anlotinib; Radiotherapy	Concomitant therapy	PFS
NCT07071103 (176)	II	ILDR plus PD-1/PD-L1 inhibitors	Concomitant therapy	ORR; DCR; PFS2; Incidence of abscopal effect
NCT05383482 (177)	I/II	Afuresertib; Nab-paclitaxel; Sintilimab; Docetaxel	Concomitant therapy	AEs; DLT; ORR

For ongoing trials or trials without publicly available results, the cited source refers to the trial registration record and supports the study design and prespecified outcome measures only; it does not indicate confirmed treatment efficacy.

### 6.2 Future directions and breakthrough points

#### 6.2.1 Individualized treatment guided by molecular typing

The significant heterogeneity of esophageal squamous cell carcinoma is a primary factor contributing to drug resistance, making the establishment of a precise molecular typing system essential for progress. The EGIC typing, developed from the JUPITER-06 study (13), incorporates multi-dimensional indicators such as ccTMB, HLA genotypes, and oncogenic variations, enabling accurate predictions of which populations will benefit from combined immunotherapy. A research team from the Chinese Academy of Medical Sciences conducted a multi-omics analysis (80) that classified esophageal squamous cell carcinoma into four distinct types: cell cycle pathway activated type (CCA), NRF2 pathway activated type (NRFA), immunosuppressive type (IS), and immunomodulatory type (IM). This classification provides a theoretical framework for tailoring personalized combination regimens for different subtypes.

The optimized application of biomarkers represents a fundamental component of personalized treatment. CD8+ T cells exhibiting high CD39 expression (144, 145) serve as a robust predictor of response to PD-1 inhibitors, surpassing traditional assessments of PD-L1 expression. Additionally, peripheral blood TRGC2+ NKT cells (146) can reliably forecast the pathological response to neoadjuvant immunochemotherapy. These markers have been validated through external cohorts and are anticipated to be integrated into clinical practice. Nevertheless, a central challenge in translating these biomarkers into clinical practice is distinguishing stable, disease-intrinsic molecular features from patient-specific and context-dependent variation across heterogeneous cohorts and treatment settings; robust, generalizable biomarkers therefore require integration into multi-omics stratification frameworks and prospective validation in independent multicenter cohorts.

#### 6.2.2 New targets and drug development

Targeting novel cell subpopulations or signaling pathways within the physical and biochemical barriers established by stromal cells in the TME can significantly enhance the penetration and efficacy of ICIs. For example, GPR116-positive pericytes activate the NF-κB pathway and promote esophageal cancer metastasis by secreting EGFL6, which binds to tumor cell integrin β1. This process induces CD8^+^ T cell exhaustion and upregulates PD-L1 expression through IL-10/TGF-β signaling. In murine models, the combination of integrin β1 inhibitors and PD-1 inhibitors markedly inhibited the progression and metastasis of the primary lesion compared to monotherapy, thereby providing a direct basis for clinical translation (147). Additionally, targeting TME-mediated immunosuppression represents a crucial strategy to overcome drug resistance. CAFs and TAMs serve as core mediators of TME immunosuppression. Recent studies indicate that in ovarian cancer models, inhibiting nicotinamide N-methyltransferase (NNMT) (148) in CAFs can reshape their epigenetic state, reduce complement activity, and decrease the recruitment of MDSCs, ultimately restoring anti-tumor immunity, which may emerge as a novel therapeutic target. In esophageal squamous cell carcinoma, NNMT has likewise been shown to act as a master metabolic regulator of epithelial-mesenchymal transition and metastasis (149). Furthermore, regarding SLC1A3-mediated glutamate metabolic crosstalk (150), inhibitors targeting glutamate transporters have progressed to preclinical research and are anticipated to reverse the conversion that leads to combined drug resistance. The combination of drugs targeting molecular alterations such as NOTCH1 mutations (151, 152) with immunotherapy has also shown synergistic effects in subgroup analyses, suggesting a new direction for addressing specific drug-resistant populations.

#### 6.2.3 Innovative applications of cell therapy

Cell therapy introduces a novel therapeutic approach to enhancing immune resistance. Researchers from the Chinese Academy of Medical Sciences found a significant deficiency of central memory T cells (TCM) in the tumor tissues of patients with esophageal squamous cell carcinoma (145). The reinfusion of peripheral blood CD62L+ TCM cells effectively eliminates autologous tumor cells, offering a promising strategy to address immune deficiencies within the TME. Investigations into CAR-T cell therapy and tumor-infiltrating lymphocyte (TIL) therapy for esophageal cancer have progressed steadily. Early clinical trials have demonstrated that CAR-T therapy combined with PD-1 inhibitors targeting antigens such as HER2 and Claudin 18.2 (153) can achieve local control. Looking ahead, optimizing tumor targeting and extending the survival of cell therapy products through gene editing technology may provide a breakthrough in the treatment of solid tumors.

#### 6.2.4 The expansion of a precision diagnosis and treatment system and model empowered by technology

In addition to the previously mentioned single-cell sequencing technology, which can dynamically monitor changes in immune cell subsets within the TME during treatment, organoid models (154, 155) may help validate the predictive value of these cell subsets and provide a platform for drug sensitivity screening. This approach enables a closed-loop transformation of “clinical problems - laboratory verification - clinical application.”

Advancing the timing of immunotherapy represents a critical research avenue for enhancing long-term survival. The ESCORT-NEO study (156) revealed that the pathological complete response rate for Camrelizumab combined with chemotherapy as neoadjuvant therapy was 3 to 6 times greater than that of chemotherapy alone, without increasing surgical risk. This finding offers the potential for downstaging and achieving radical cure in patients with locally advanced disease. Furthermore, “organ-preserving therapy” has emerged as a prominent topic of interest. Numerous studies, both domestically and internationally, including preSINO (157) and SANO (158), have investigated active surveillance after neoadjuvant chemoradiotherapy to extend survival while maintaining esophageal function. Concurrently, the prediction and management of immune-related adverse events (irAE), along with comprehensive nutritional support, are essential components that can significantly enhance patients’ treatment tolerance and indirectly mitigate the risk of drug resistance associated with treatment interruptions.

### 6.3 Research limitations and clinical translation challenges

Despite significant advances in understanding resistance mechanisms, several limitations must be acknowledged to guide future research and temper clinical expectations. The majority of mechanistic insights derive from preclinical models, such as cell lines and patient-derived xenografts, which may not fully capture the complexity of the TME and inter-patient heterogeneity, and most studies remain cross-sectional rather than longitudinal, limiting our understanding of how resistance evolves dynamically during treatment; consequently, large-scale prospective validation of mechanistically derived biomarkers remains scarce. Regarding combination therapies, while regimens such as ICIs plus anti-angiogenics or ADCs show promise, additive or synergistic toxicities and the challenge of determining optimal treatment sequencing are major concerns—dual ICIs (e.g.,PD-1 + CTLA-4) are associated with grade ≥3 irAEs in up to 35–40% of patients, including potentially fatal colitis and myocarditis, and combining ADCs with ICIs may increase the risk of pneumonitis and peripheral neuropathy, necessitating rigorous patient monitoring and the development of predictive toxicity biomarkers. Biomarker implementation also faces multiple bottlenecks: lack of standardized detection methods and cutoffs across institutions, high cost and limited accessibility of multi-omics assays, intratumoral heterogeneity requiring multiple biopsies, and limited validation in prospective multicenter cohorts, meaning that biomarker-guided patient stratification will remain largely investigational until these issues are resolved. Furthermore, as noted throughout this review, the vast majority of mechanistic evidence pertains to ESCC, whereas EAC—the predominant subtype in Western populations—remains understudied; extrapolating ESCC-derived findings to EAC may be inappropriate given their distinct etiologies, TME compositions, and metabolic profiles. Thus, dedicated studies in EAC are urgently needed to determine whether similar resistance mechanisms operate and whether combination strategies can be directly translated. Addressing these limitations will require coordinated efforts involving multi-institutional consortia, standardized protocols for biomarker testing, and the incorporation of real-world evidence alongside clinical trials. Future research should focus on developing more sophisticated models, such as patient-derived organoids (154, 155), leveraging spatial omics to understand resistance *in situ* (61), and designing clinical trials that incorporate robust biomarker-driven patient stratification to accelerate the translation of these findings into clinical practice.

## 7 Conclusion

The resistance of esophageal cancer to immunotherapy presents a significant challenge, arising from the intricate interplay of dynamic TME immunosuppression, substantial metabolic reprogramming, complex epigenetic regulation, and compensatory activation of alternative immune checkpoints. To overcome this drug resistance, it is essential to develop rational combination strategies that concurrently target multiple facets of immune escape, thereby enhancing clinical efficacy and achieving durable responses. Future research should prioritize the identification of reliable predictive biomarkers, the development of novel therapeutic agents aimed at these drug resistance mechanisms, and the design of innovative clinical trials to validate these multimodal approaches. Such efforts are crucial for improving the prognosis and quality of life for patients with esophageal cancer. Although the path to surmounting drug resistance in immunotherapy for esophageal cancer is intricate, our advancing understanding of these molecular mechanisms brings us closer to the prospect of more effective and personalized treatments.
